# Catamenial loss of botulinum toxin efficacy successfully managed with tamoxifen: A case report

**DOI:** 10.1016/j.jdcr.2026.06.048

**Published:** 2026-06-27

**Authors:** Cristelle Obeid, Simona Abou Tayeh, Georgio Chidiac, Nakhle Ayoub

**Affiliations:** a School of Medicine and Medical Sciences, Holy Spirit University of Kaslik, Jounieh, Lebanon; b Department of Dermatology, Notre Dame des Secours University Hospital, Byblos, Lebanon; c Department of Dermatology, Saint George Hospital University Medical Center, Saint George University of Beirut, Beirut, Lebanon; d Dermatology Department, Hôtel-Dieu de France, Beirut

**Keywords:** botulinum toxin, botulinum toxin type A, catamenial phenomenon, hormone-related treatment failure, neuromodulator resistance, tamoxifen

## Introduction

Botulinum toxin type A (BoNT-A) injections represent the most common nonsurgical aesthetic procedure worldwide, with the market valued at over $4 billion annually and growing at ∼12% per year. The widespread adoption reflects societal concern with facial aging and its psychosocial impact on quality of life and self-esteem. The reported duration of clinical effect for BoNT-A in cosmetic applications is typically between 3 and 6 months.^1^ However, several factors may affect the longevity of its effects. These include the injection technique, disruption of the cold chain during storage or transport, compromised toxin integrity, improper dilution, inadequate dosing, and the development of resistance.^2^ The potential influence of hormonal fluctuations on treatment efficacy has not been well explored. Sex hormones are known to modulate neuromuscular transmission and neural excitability, raising the possibility that hormonal changes could influence BoNT-A response.^3^^,^^4^

Here, we report an uncommon case of a patient who consistently experienced a marked loss of BoNT-A efficacy coinciding with the onset of her menses.

## Case report

A 45-year-old woman presented with a history of abrupt recurrent loss of BoNT-A effect for upper facial rhytids coinciding with the onset of menstruation. Her past medical history was unremarkable. She reported regular menses, was a chronic smoker since 2001, and had no history of pregnancies.

She reported undergoing her first aboBoNT-A (Dysport) session in July 2019, achieving early clinical response showing at day 4 with maximal effect at day 14 (Supplementary Video 1, available via Mendeley at https://data.mendeley.com/datasets/3h47mt9fzs/1). However, 3 days after the onset of her menses, corresponding to 21 days after her injection session, she noted a complete loss of effect (Supplementary Video 2, available via Mendeley at https://data.mendeley.com/datasets/3h47mt9fzs/1).

A second session was performed 4 months later, in November 2019, with onabotulinumtoxinA (Botox). Again, the initial response was satisfactory but dissipated abruptly on the third day of menstruation, which was 24 days after her BoNT-A session.

She subsequently underwent 2 additional sessions, each resulting in the same pattern coinciding with the onset of menstruation. All sessions performed consistently included injections targeted at the glabellar complex (corrugator supercilii and procerus muscles), frontalis, and lateral canthal regions, using a standard aesthetic injection technique. The exact dosing and dilution parameters were not available.

Because she consistently achieved the expected onset and peak response with 3 different preparations of BoNT-A, botulinum toxin resistance was considered unlikely. A possible hormonally mediated mechanism was hypothesized, and a trial of tamoxifen was suggested to the patient. The potential benefits and possible adverse effects were discussed, and informed consent was obtained prior to initiation of therapy.

Tamoxifen 20 mg was administered daily for 1 week prior to each menstrual cycle (last week of the luteal phase) and discontinued on the first day of menses. The patient received onabotulinumtoxinA (Botox) on day 2 of her menstrual cycle. Visible reduction of facial rhytids was observed as expected and maintained with effects persisting for 5.5 months (Supplementary Video 3, available via Mendeley at https://data.mendeley.com/datasets/3h47mt9fzs/1). After discontinuing tamoxifen, a subsequent Dysport injection during the menstrual phase of her cycle resulted in a reproducible loss of effect. A third treatment cycle, administered while tamoxifen was resumed, demonstrated sustained efficacy for up to 6 months. These observations suggest a previously unreported interaction between menstrual-cycle–related physiologic changes and botulinum toxin pharmacodynamics or neuromuscular sensitivity, with tamoxifen potentially mitigating this cyclical loss of effect. A structured timeline summarizing the temporal association between menstrual cycle phases, BoNT-A response, and therapeutic interventions is presented in Table I. As shown, a reproducible reduction in efficacy was observed after menses, which was attenuated following initiation of tamoxifen.

**Table I:** Timeline of clinical course, menstrual cycle correlation, and treatment response

Time point	Menstrual phase	Intervention	BoNT-A response	Clinical observation
Mo 0	Late follicular phase	BoNT-A injection	Good response (Video 1)	Expected reduction in dynamic rhytids
Mo 1 (21 d after her first session)	Early follicular phase (d 3 of the menstrual cycle)	None	Complete loss of effect (Video 2)	Early return of muscle activity and wrinkles
Mo 4	Late follicular phase	Repeat BoNT-A	Good response	Restoration of effect
Mo 5 (24 d after her session)	Early follicular phase (d 3 of the menstrual cycle)	None	Recurrent reduced efficacy	Reproducible cyclical pattern
Mo 6	Tamoxifen initiated			
Mo 7	At d 2 of the menstrual cycle (early follicular phase)	BoNT-A ± tamoxifen	Sustained response for 5.5 mo (Video 3)	Improved durability
Mo 8	Luteal	Discontinued tamoxifen	Complete loss of effect after menses	Early return of muscle activity and wrinkles
Mo 9	Luteal	BoNT-A ± tamoxifen	Stable response for 6 mo	No significant catamenial variation

*BoNT-A*, Botulinum toxin type A.

## Discussion

This is not a case of primary or secondary resistance to botulinum toxin treatment because the patient experienced the full clinical action of BoNT-A immediately after the injection and then lost it abruptly at the onset of her menstruation.^5^ Although neutralizing antibodies were not formally assessed, the cyclical pattern of reduced efficacy argues against immunogenic resistance, which typically presents as persistent secondary nonresponse. The results could not be attributed to one single formulation of BoNT-A because 3 different formulations were used with the same outcome. The success of the therapeutic trial with daily oral tamoxifen suggests a potential relationship between menstrual hormones and BoNT-A responsiveness because the treatment appeared to maintain the efficacy of BoNT-A injections.

Tamoxifen is a selective estrogen receptor (ER) modulator that acts as an estrogen antagonist in breast tissue and as an estrogen agonist in other tissues, such as endometrium. Its primary mode of action is competitive inhibition of estrogen binding to ER, thereby reducing ER-dependent gene transcription. However, tamoxifen exerts indirect effects on progesterone receptor expression and activity through its modulation of ER signaling. Tamoxifen frequently affects menstrual function; the most common menstrual disturbance is amenorrhea.^6^

Cyclic variations in hormones during the normal menstrual cycle are known to influence the course of several diseases. For instance, for dermatologic conditions, the most reported primary catamenial dermatoses are autoimmune progesterone dermatitis and autoimmune estrogen dermatitis. Additionally, the most commonly reported chronic skin disorders exacerbated by menses are atopic dermatitis, psoriasis, and acne vulgaris.^7^ The catamenial fluctuations of these diseases are explained mainly by hormonal-receptor-tissue activation and known modulation of the innate immune system.^8^ Tamoxifen has been reported in the medical literature as an effective treatment for estrogen dermatitis, a rare condition characterized by cyclic or premenstrual flares of urticaria, eczema, papulovesicular eruptions, and pruritus. Tamoxifen has also demonstrated efficacy in autoimmune progesterone dermatitis, suggesting its utility in hormonally mediated dermatoses.^9^ Nevertheless, tamoxifen has a capacity to antagonize ER activity in skin/immune cells, disrupting the hormonal amplification of dendritic cell–mediated inflammation as previously described in estrogen dermatitis. This could explain the possible similar shared mechanism of action in our current case.^10^

Furthermore, BoNT-A works by cleaving the Soluble N-ethylmaleimide-sensitive factor attachment protein receptor, SNAP-25, in the presynaptic terminal, preventing acetylcholine release at the neuromuscular junction of the targeted muscle. ERs are present in motor neurons and muscle fibers and modulate acetylcholine receptor stability and neurotransmitter release, influencing neuromuscular transmission.^3^ Moreover, we hypothesized that a transient surge of progesterone in the luteal phase could theoretically counteract BoNT-A–mediated blockade and induce a perceptible drop in clinical efficacy of the toxin in the perimenstrual period. Cytotoxic and helper T cells as well as macrophages and monocytes, express ERs. The activation of estrogen pathways is associated with the expression of toll-like receptors. Toll-like receptor upregulation lowers the innate immune threshold and enhances immune response when estrogen levels are elevated, which might explain why certain autoimmune conditions fluctuate in severity during menstruation.^7^

In this case, tamoxifen therapy led to a reversal of the catamenial loss of botulinum toxin efficacy, providing clinical evidence of a potential hormonal influence. Future research should include prospective and randomized studies assessing BoNT-A efficacy across menstrual cycle phases and evaluating the potential role of hormonal-modulation strategies. Additionally, larger studies are needed to determine the reproducibility and clinical relevance of catamenial variation in BoNT-A response.

The observed association between menstrual cycle phase and botulinum toxin response remains hypothetical because there is no direct experimental evidence linking sex-hormone fluctuations to BoNT-A pharmacodynamics. Long-term management considerations are also important, particularly given the potential adverse effects of tamoxifen, highlighting that patient safety must remain the primary concern when considering off-label interventions for aesthetic indications. Neutralizing antibodies against BoNT-A were not formally assessed, which limits definitive exclusion of immunogenic resistance.

We report a single case of an unusual catamenial loss of botulinum toxin effect for upper facial rhytids, treated successfully with the use of tamoxifen, identifying a possible hormonally mediated mechanism. This case suggests a possible role for hormonal modulation in neurotoxin response and emphasizes the need for clinicians to consider cyclical patterns when evaluating apparent treatment resistance.

## Conflicts of interest

None disclosed.
